# Coexistence of Hypothyroidism and Vitiligo Vulgaris: A Case Report

**DOI:** 10.7759/cureus.91669

**Published:** 2025-09-05

**Authors:** Pankaj Kumar, Md Jawed Akhtar

**Affiliations:** 1 Department of Nuclear Medicine, All India Institute of Medical Sciences, Patna, Patna, IND; 2 Department of Anatomy, Indira Gandhi Institute of Medical Sciences, Patna, IND

**Keywords:** autoimmune thyroiditis, depigmented patch, hypothyroidism, levothyroxine, vitiligo

## Abstract

Vitiligo vulgaris is a chronic depigmenting disorder commonly linked to autoimmune diseases, particularly autoimmune thyroid conditions. We report a case of a 34-year-old female patient with well-controlled primary hypothyroidism for four years who, during a routine follow-up at the outpatient thyroid clinic in the nuclear medicine department of a tertiary care centre, noted the recent appearance of asymptomatic depigmented patches on her right forearm and left leg. Dermatological examination confirmed a diagnosis of vitiligo vulgaris. With no systemic symptoms and normal thyroid function, the coexistence suggests a shared autoimmune predisposition. This case highlights the importance of routine follow-up in identifying emerging autoimmune features and reinforces the value of a multidisciplinary approach in managing chronic autoimmune disorders.

## Introduction

Autoimmune diseases often cluster within the same individual, reflecting shared genetic, immunological, and environmental predispositions. Among these, autoimmune thyroid disorders (AITD) are the most prevalent, with hypothyroidism - most commonly due to Hashimoto’s thyroiditis - representing a major clinical entity. Patients diagnosed with hypothyroidism frequently develop additional autoimmune conditions during the course of their illness, underscoring the systemic nature of immune dysregulation. Vitiligo vulgaris, an acquired depigmenting disorder characterized by the selective destruction of melanocytes, is one such condition that has been closely associated with AITD [1-5]. While vitiligo is not life-threatening, its visible manifestations often carry significant psychosocial consequences, affecting self-image and quality of life. The coexistence of hypothyroidism and vitiligo not only highlights the interplay between organ-specific and systemic autoimmunity but also raises important questions regarding shared pathophysiological mechanisms, early recognition, and comprehensive patient care. In this case report, we present a case of a young Indian female patient with well-controlled hypothyroidism who later developed vitiligo, emphasizing the need for heightened clinical vigilance in monitoring for additional autoimmune manifestations. This case underscores the importance of considering autoimmune diseases as a spectrum rather than isolated entities, with implications for long-term management and patient well-being.

## Case presentation

A 34-year-old married female patient with primary hypothyroidism, diagnosed five years ago, presented for a routine follow-up at the outpatient thyroid clinic in the nuclear medicine department of a tertiary care center. She was diagnosed with hypothyroidism during routine first-trimester thyroid screening and found to have elevated serum thyroid-stimulating hormone (TSH) level of 10.2 µIU/mL (N: 0.35-5.5) and low free triiodothyronine (FT3), low free thyroxine (FT4), and a significantly elevated anti-thyroid peroxidase (TPO) antibody level of >1,300 IU/mL (N: <60). She was initiated on a daily dose of 50 mcg oral levothyroxine replacement therapy. Thyroid function tests (TFTs) returned to normal levels after a month of initiation of therapy, and she delivered a healthy baby with normal TFTs. The patient’s TFTs had remained within normal limits during recent follow-ups, and she did not report any new symptoms suggestive of thyroid dysfunction.

At this visit, her laboratory findings revealed normal thyroid function test results (Table 1). Other investigations, including serum calcium, hemoglobin, white blood cell (WBC) count, fasting and postprandial plasma glucose, total protein, total bilirubin, serum creatinine, and total cholesterol, were all within normal limits. All parameters were normal, except for serum glutamic pyruvic transaminase (SGPT), which was noted to be slightly elevated (Table 1).

**Table 1:** Recent investigation report of the 34-year-old female patient with hypothyroidism who noticed depigmented patches on the right forearm and left leg

Test	Result	Reference range
Serum thyroid-stimulating hormone (TSH)	2.5 µIU/mL	0.35 - 5.5 µIU/mL
Serum free triiodothyronine (FT3)	5.3 pmol/L	3.5 - 6.5 pmol/L
Serum free thyroxine (FT4)	15.3 pmol/L	11.5 - 22.7 pmol/L
Serum calcium	9 mg/dL	8.6 - 10 mg/dL
Hemoglobin	11.6 g/dL	12 - 15 g/dL
White blood cell (WBC) count	8,520 /mm³	4,000 - 10,000 /mm³
Blood glucose fasting	95 mg/dL	75 - 110 mg/dL
Blood glucose post-prandial	101 mg/dL	<140 mg/dL
Total protein	7.3 g/dL	6.4 - 8.3 g/dL
Total bilirubin	0.6 mg/dL	0.3 - 1.2 mg/dL
Serum glutamic pyruvic transaminase (SGPT)	30.9 U/L	10 - 28 U/L
Serum glutamic oxaloacetic transaminase (SGOT)	19 U/L	<31 U/L
Serum creatinine	0.6 mg/dL	0.7 - 1.3 mg/dL
Total cholesterol	165 mg/dL	<200 mg/dL

**Figure 1:**
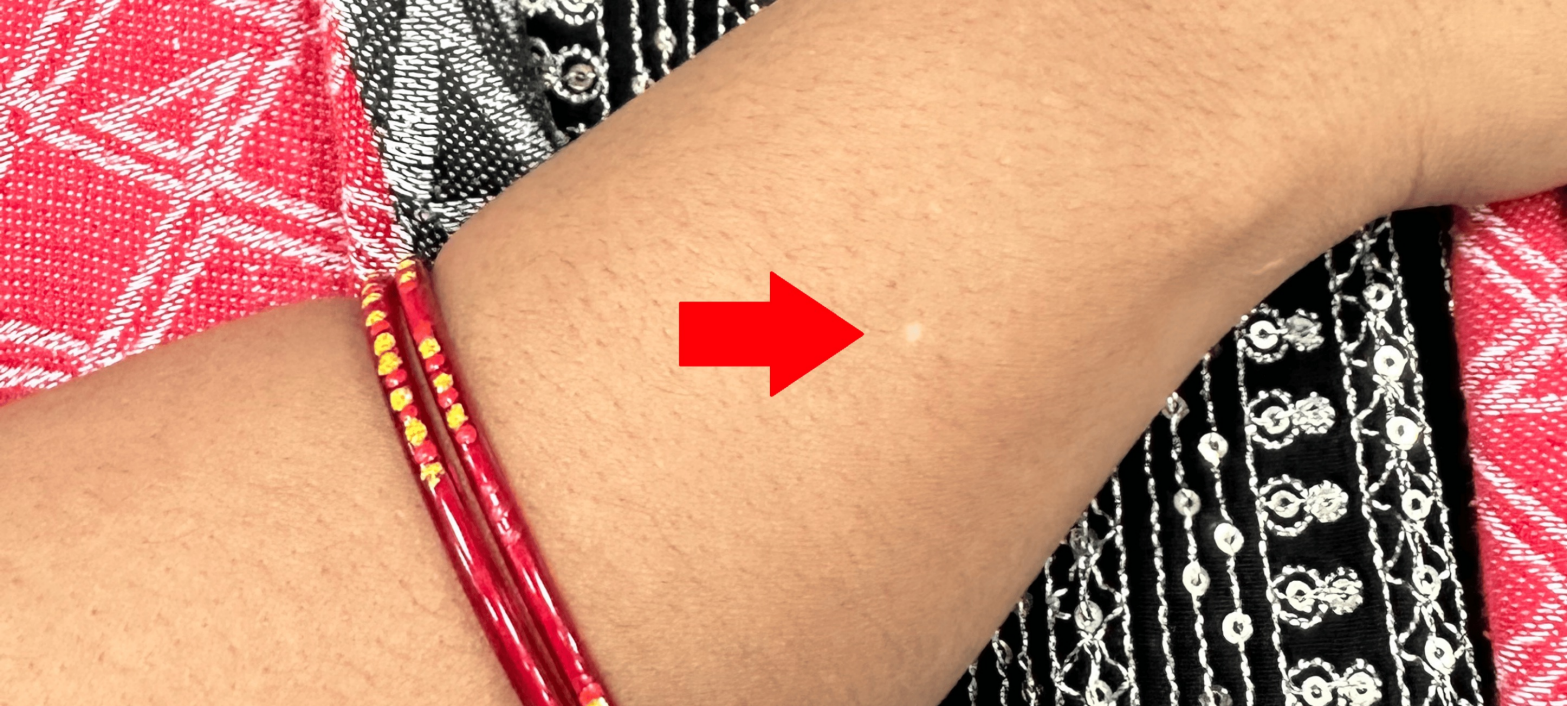
Close view of the depigmented patch (red arrow) on the dorsal aspect of the right forearm of the patient

**Figure 2:**
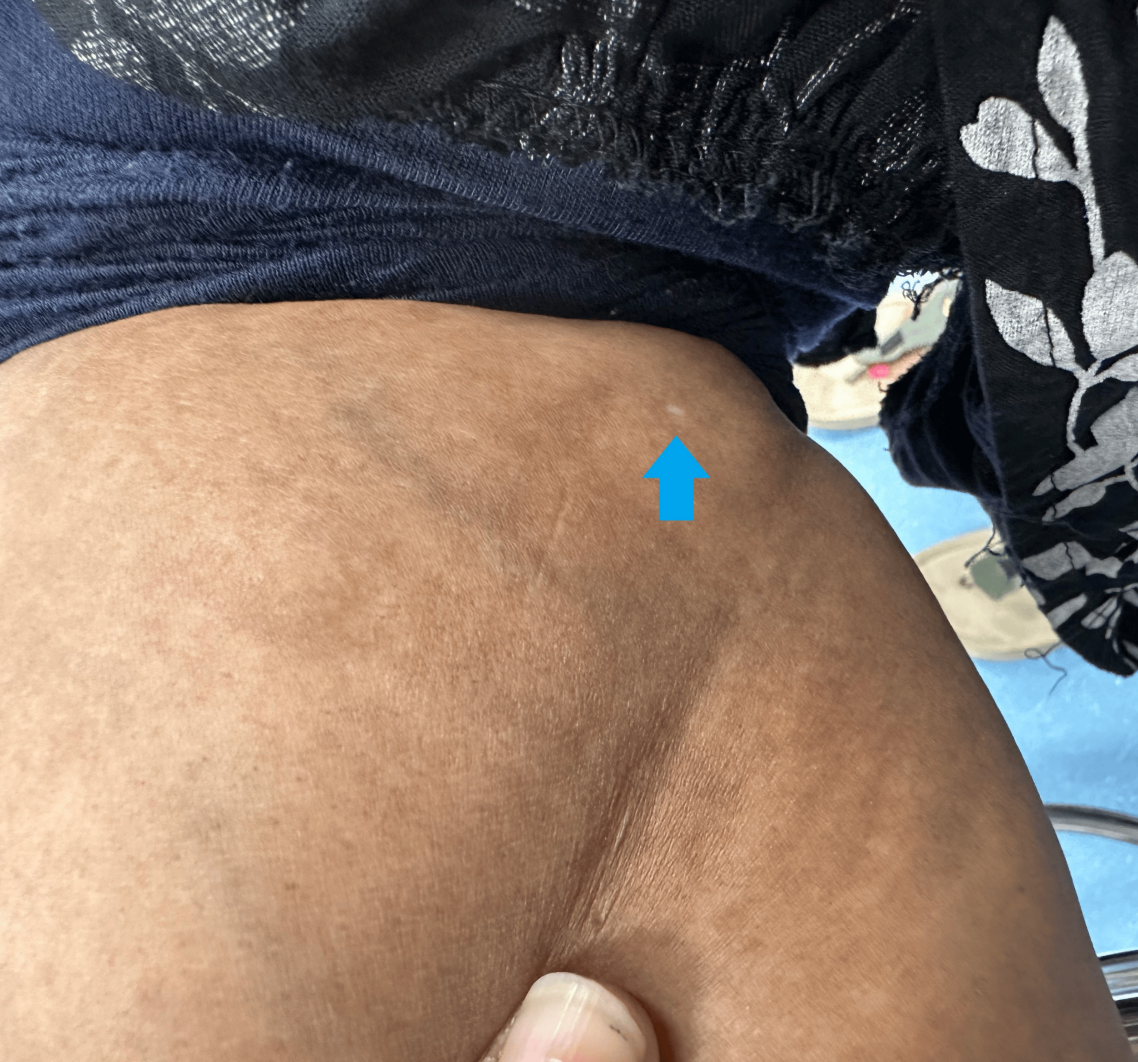
Depigmented patch (blue arrow) on the posterior aspect of the left leg of the patient

During the consultation, the patient mentioned the recent appearance of skin changes that had developed over the past three months. She described noticing a small, round, depigmented patch on the dorsal aspect of her right forearm (Figure 1), followed by a similar lesion on the posterior aspect of her left leg, just below the knee (Figure 2). These patches were asymptomatic, with no itching, pain, or scaling, but she expressed concern about their progression.

On dermatological examination, well-demarcated, non-scaly depigmented macules consistent with vitiligo vulgaris were observed. There were no signs of inflammation or secondary changes. Her physical examination revealed no additional significant findings, with stable vital signs and a regular pulse rate of 72 beats per minute. No additional skin or mucosal lesions were identified.

Given her history of AITD and the new onset of vitiligo, an autoimmune etiology was considered likely. Although she had no reported family history of autoimmune disorders, the co-occurrence of these conditions pointed to an underlying autoimmune predisposition.

The patient was counseled regarding the nature of vitiligo and its known association with AITD. She was referred to the dermatology department for further assessment and management, which included topical treatment and photoprotection measures. Regular monitoring of her thyroid function and periodic skin evaluations were advised to track disease progression and detect any new lesions.

## Discussion

This case underscores the need for vigilance in recognizing subtle clinical changes, even during routine follow-ups in patients with stable chronic conditions. The patient, a 34-year-old female with well-controlled primary hypothyroidism, presented without any new complaints related to her thyroid health. Her TFTs remained within the normal range, and there were no symptoms to suggest disease progression or imbalance. However, her mention of new skin changes, specifically asymptomatic depigmented patches, prompted a clinical assessment, which confirmed vitiligo vulgaris. Vitiligo is a chronic skin disorder characterized by the progressive disappearance of melanocytes, the cells responsible for skin pigmentation. This results in clearly defined areas of depigmented skin. Although the exact cause is not yet fully understood, growing evidence suggests that autoimmune processes are a key contributing factor in many cases [6].

Autoimmune conditions often do not appear in isolation. In fact, vitiligo is known to be associated with various autoimmune disorders, such as thyroid dysfunction, insulin-dependent diabetes mellitus, Addison’s disease, alopecia areata, etc. Autoimmune thyroiditis is one of the most commonly associated conditions observed in patients with vitiligo. Reported co-occurrence rates vary between 15% and 30%, underscoring a significant overlap in their autoimmune basis. These estimates are largely derived from case-control and cross-sectional studies conducted in dermatology and endocrinology clinic populations. In these studies, vitiligo was typically diagnosed on the basis of characteristic clinical features, while thyroid dysfunction was confirmed through laboratory evaluation, including TFTs and the detection of thyroid autoantibodies. Together, these findings highlight the importance of routine thyroid screening in individuals with vitiligo to ensure early detection and timely management of associated thyroid disease [7-15]. These findings in this patient are significant, not just because of the skin involvement, but because of their broader implications. The co-occurrence of vitiligo in a patient with AITD suggests a shared autoimmune basis (possibly T-cell-mediated destruction or shared genetic loci like human leukocyte antigen (HLA) associations) [16].

In this patient, although there was no family history of autoimmune disease, the presence of both conditions raises the possibility of a broader, underlying autoimmune tendency. Importantly, her skin lesions were asymptomatic and might easily have been overlooked or dismissed without further assessment. This case underlines the need for clinicians to maintain a high index of suspicion for related autoimmune manifestations, even when primary disease management appears stable. The patient was appropriately counseled about the nature of vitiligo, its course, and its relationship with AITD. Referral to dermatology enabled targeted treatment with topical agents and education on photoprotection [17]. Ongoing monitoring of both her thyroid status and skin condition was recommended to ensure timely detection of any progression.

Overall, this case serves as a reminder that routine check-ups offer opportunities not only to monitor existing conditions but also to detect new and potentially related developments. A multidisciplinary approach and open patient communication are essential in managing chronic autoimmune conditions and addressing the full scope of their manifestations.

## Conclusions

This case illustrates how routine follow-ups can uncover subtle signs of evolving autoimmune conditions, even when existing diseases appear well-controlled. It underscores the value of listening closely to patient concerns and adopting a collaborative, multidisciplinary approach to ensure early recognition and comprehensive care of interconnected autoimmune disorders.

## References

[1] Taïeb A, Picardo M (2009). Vitiligo. N Engl J Med.

[2] Bergqvist C, Ezzedine K (2020). Vitiligo: a review. Dermatology.

[3] Pandve HT (2008). Vitiligo: is it just a dermatological disorder?. Indian J Dermatol.

[4] Baldini E, Odorisio T, Sorrenti S (2017). Vitiligo and autoimmune thyroid disorders. Front Endocrinol (Lausanne).

[5] Prashant P, Garg R, Bansal P, Praveen S (2023). Thyroid autoimmunity in vitiligo: a case-control study. Cureus.

[6] Rodrigues M, Ezzedine K, Hamzavi I, Pandya AG, Harris JE (2017). New discoveries in the pathogenesis and classification of vitiligo. J Am Acad Dermatol.

[7] Spritz RA (2007). The genetics of generalized vitiligo and associated autoimmune diseases. Pigment Cell Res.

[8] Malik S, Cohen PR (2021). Vitiligo-associated autoimmune disorders: a woman with vitiligo and incipient hypothyroidism. Cureus.

[9] Chen Y, Zhang Y, Liu W, Huang X, Luo X, Wang H (2024). The causal relationship between vitiligo and autoimmune thyroid diseases: a bidirectional two-sample Mendelian randomization analysis. Skin Res Technol.

[10] Khiangte L, Lalrindik C (2023). Study of thyroid disorders in vitiligo. J Family Med Prim Care.

[11] Gopal KV, Rao GR, Kumar YH (2014). Increased prevalence of thyroid dysfunction and diabetes mellitus in Indian vitiligo patients: a case-control study. Indian Dermatol Online J.

[12] Chivu AM, Bălășescu E, Pandia LD (2022). Vitiligo-thyroid disease association: when, in whom, and why should it be suspected? A systematic review. J Pers Med.

[13] Sedighe M, Gholamhossein G (2008). Thyroid dysfunction and thyroid antibodies in Iranian patients with vitiligo. Indian J Dermatol.

[14] Abuhalimeh RM, Alshmrani LS, Abdullah N (2024). Updates on the association between vitiligo and thyroid diseases: a systematic review. Cureus.

[15] Upadhya S, Andrade MJ, Shukla V, Rao R, Satyamoorthy K (2025). Genetic and immune dysregulation in vitiligo: insights into autoimmune mechanisms and disease pathogenesis. Autoimmun Rev.

[16] Spritz RA (2010). Shared genetic relationships underlying generalized vitiligo and autoimmune thyroid disease. Thyroid.

[17] Frisoli ML, Essien K, Harris JE (2020). Vitiligo: mechanisms of pathogenesis and treatment. Annu Rev Immunol.

